# Bracketing phenogenotypic limits of mammalian hybridization

**DOI:** 10.1098/rsos.180903

**Published:** 2018-11-28

**Authors:** Yoland Savriama, Mia Valtonen, Juhana I. Kammonen, Pasi Rastas, Olli-Pekka Smolander, Annina Lyyski, Teemu J. Häkkinen, Ian J. Corfe, Sylvain Gerber, Isaac Salazar-Ciudad, Lars Paulin, Liisa Holm, Ari Löytynoja, Petri Auvinen, Jukka Jernvall

**Affiliations:** 1 Developmental Biology Program, Institute of Biotechnology, University of Helsinki, PO Box 56, 00014 Helsinki, Finland; 2 Genome Biology Program, Institute of Biotechnology, University of Helsinki, PO Box 56, 00014 Helsinki, Finland; 3 Faculty of Biological and Environmental Sciences, University of Helsinki, PO Box 56, 00014 Helsinki, Finland; 4 Department of Environmental and Biological Sciences, University of Eastern Finland, PO Box 111, 80101 Joensuu, Finland; 5 Institut Systématique Evolution Biodiversité (ISYEB), Muséum national d'Histoire naturelle, CNRS, Sorbonne Université, EPHE, 45 rue Buffon, CP 50, 75005 Paris, France; 6 Departament de Genètica i Microbiologia, Universitat Autònoma de Barcelona, 08193 Cerdanyola del Vallès, Spain

**Keywords:** species hybridization, introgression, developmental conservation, disparity, morphology, dental

## Abstract

An increasing number of mammalian species have been shown to have a history of hybridization and introgression based on genetic analyses. Only relatively few fossils, however, preserve genetic material, and morphology must be used to identify the species and determine whether morphologically intermediate fossils could represent hybrids. Because dental and cranial fossils are typically the key body parts studied in mammalian palaeontology, here we bracket the potential for phenotypically extreme hybridizations by examining uniquely preserved cranio-dental material of a captive hybrid between grey and ringed seals. We analysed how distinct these species are genetically and morphologically, how easy it is to identify the hybrids using morphology and whether comparable hybridizations happen in the wild. We show that the genetic distance between these species is more than twice the modern human–Neanderthal distance, but still within that of morphologically similar species pairs known to hybridize. By contrast, morphological and developmental analyses show grey and ringed seals to be highly disparate, and that the hybrid is a predictable intermediate. Genetic analyses of the parent populations reveal introgression in the wild, suggesting that grey–ringed seal hybridization is not limited to captivity. Taken together, we postulate that there is considerable potential for mammalian hybridization between phenotypically disparate taxa.

## 1. Introduction

Although hybridization has been extensively examined in the context of speciation, the role of interbreeding leading to introgression and admixture of phenotypic traits is attracting increasing attention [1–7]. In studies of human evolution, genetic evidence has implicated introgression between different lineages of *Homo* [8,9], and an increasing number of mammalian fossils are suggested to retain signs of interbreeding by palaeogenomic studies [10–12]. Hybridization can complicate the assignment of fossil specimens to specific species [4,13–15]. Even if hybrids between morphologically dissimilar species have reduced fertility, they may still be preserved in the fossil record, especially if hybridization is fairly common as in active hybrid zones (e.g. [2,4,16,17]). Domesticated mammals can show large differences in features such as size and skull shape, but other adaptively and taxonomically important features such as dentitions remain largely monotypic among domestic breeds. Therefore, to assess the maximum morphological range of potential hybridization in evolution, analyses of hybrids between morphologically disparate wild taxa are required. One such hybridization, with uniquely preserved cranial and dental material, has been reported to have occurred in Stockholm zoo in 1929 between two mammalian species belonging to different genera; the grey seal (*Halichoerus grypus*) and the ringed seal (*Pusa hispida*) [18]. This incident raises questions as to how distinct these species are genetically, how distinct these species are phenotypically, how easy it is to identify the hybrids using morphology and whether comparable hybridizations happen in the wild. Addressing all these questions together allows one to estimate the ‘hybrid bracket’, or the overall potential for hybridization in mammalian evolution.

## 2. Results and discussion

### 2.1. Validating the seal hybrid and the genetic context of the hybridization

The seal born in 1929 was immediately concluded to be a hybrid because at the time the seal pond housed only three adult seals: two male grey seals and one female ringed seal (electronic supplementary material, figure S1*a*,*b*). The newborn was found dead and although no malformations were reported [18], both biological and husbandry-related causes of death remain a possibility. First, to verify the hybridization and validate the identity of the museum specimen, we sequenced its genome (see Material and methods; electronic supplementary material, table S1) together with the genomes of its parental species, Baltic grey seals (*n* = 10) and Baltic ringed seals (*n* = 9) (see Material and Methods). A genetic admixture analysis shows that the hybrid shares roughly 50% of its genome with both species, confirming that it is indeed a hybrid between the grey and the ringed seal (electronic supplementary material, figure S1*c*).

The exact phylogenetic position and distinctiveness of the grey seal in relation to the ringed seal and other related taxa has been problematic [19–21], leaving open the question how genetically distinct the species really are. To approximate the genetic context of the seal hybridization, we computed a genome-wide estimate of neutral genetic distance between grey and ringed seals (see Material and methods), and contrasted this with species pairs well known to hybridize; lion–tiger (*Panthera leo*–*P. tigris*) and domestic donkey–horse (*Equus asinus*–*E. ferus*) (see Material and methods; electronic supplementary material, table S2). Lions and tigers readily hybridize in captivity [22], and as both they and seals are members of Carnivora, provide an appropriate comparison. Whereas these carnivoran species-contrasts have the same number of chromosomes (2*n* = 32 in seals [23,24] and 2*n* = 38 in felids [22]), donkey and horse differ in their chromosome number and their hybrids, known as mules and hinnies, are generally infertile [25]. To place the results in the context of *Homo* lineage, human and Neanderthal were also included in the analysis, as were some additional outgroups (electronic supplementary material, table S2).

**Figure 1.**
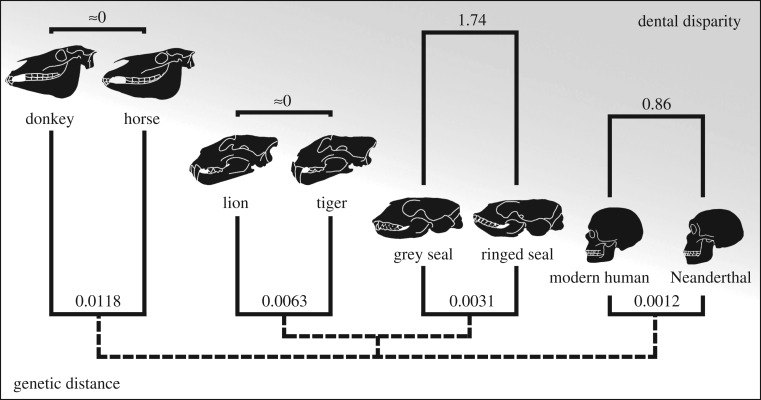
Bracketing the genetic and phenotypic distances of hybridizing taxa. Donkey–horse and lion–tiger genetic distances (substitutions per fourfold degenerate sites) are longer, and modern human–Neanderthal distance is shorter than the grey–ringed seal distance. Of the hybridizing species pairs, only grey and ringed seals are in different genera and have the most disparate dentitions (mean cusp number/tooth difference). Whereas domesticated mammals, such as dogs, can also differ in their cranial morphology, their dentitions remain relatively monotypic. Pairwise distances were calculated using fourfold degenerate sites of 4045 orthologous genes and cusp number differences are for lower postcanine teeth. For details, see Material and methods and electronic supplementary material, tables S3 and S7. The crania are not to scale.

To obtain a robust proxy of neutral sequence evolution, we used the fourfold degenerate sites of 4045 protein-coding genes, present as single copy in each species (see Material and methods). Analyses of 623 391 fourfold degenerate sites from genes orthologous for all the eight species show that the grey–ringed seal genetic distance is roughly 49% and 26% of the lion–tiger and donkey–horse distances, respectively (figure 1; electronic supplementary material, table S3). These distances appear robust when larger or smaller sets of species are compared (electronic supplementary material, tables S4 and S5). The comparatively short genetic distance between the grey and ringed seals is noteworthy because, unlike in the hybridizing lion–tiger and donkey–horse comparisons, the dental and cranial morphologies of the two seal species have historically warranted a genus-level distinction in contrast to the species-level distinctions of the lion–tiger and donkey–horse pairs.

Even though the grey–ringed seal genetic distance is shorter than the carnivore and perissodactyl contrasts (figure 1), it is still considerably longer than the hominin contrast. Compared to the modern human–Neanderthal distance, the grey–ringed seal distance is roughly two and a half times greater (258%; electronic supplementary material, table S3), suggesting that many morphologically distinct fossil hominins are within the genetic hybrid bracket. Next, we analysed the dental and cranial distinctiveness of the two seal species and their hybrid. These phenotypic structures are the key features in many paleontological studies.

### 2.2. Analysing the teeth of the hybrid

**Figure 2.**
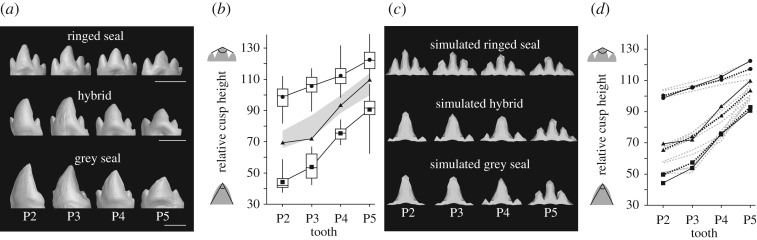
Teeth of the hybrid are morphologically and developmentally intermediate. (*a*) The hybrid has the large central cusps of the grey seal together with relatively prominent accessory cusps of the ringed seals. (*b*) The top-cusp angles of the hybrid (triangles) are intermediate between the ringed (circles) and grey (squares) seals. Grey area marks 80% of the angles averaged between each grey–ringed seal pair (electronic supplementary material, figure S3). Boxes enclose 50% of observations; the median and mean are indicated with a horizontal bar and circle or square, respectively, and whiskers denote range. (*c*) Simulated ringed and grey seal tooth rows that were manually matched to the mean real shapes and the simulated hybrid that was obtained by averaging the values of the parents. (*d*) The top-cusp angles show that the simulated hybrid teeth (triangles, black dashed line) are intermediate between the simulated grey (squares, black dashed line) and ringed (circles, black dashed line) seal teeth and comparable to the real hybrid teeth (triangles, black solid line). Grey dashed lines show hybrid simulation in which maternal or paternal values were tested for each parameter (electronic supplementary material, figure S4 and table S10). Teeth have been mirrored if needed to show left buccal views. Scale bars, 5 mm.

Mammalian tooth shape is fully formed prior to function with no remodelling other than wear after mineralization, thereby providing relatively direct information about development. In addition, seals have vestigial deciduous dentitions and are born with an erupting permanent dentition. Even though the newborn hybrid was found dead, the precocious state of seal dental development allows us to compare the hybrid with adult seals (electronic supplementary material, figure S1). The original description of the hybrid by Lönnberg [18], while detailed, was not quantitative and therefore we three-dimensionally reconstructed the hybrid dentition from microCT scans (see Material and Methods, figure 2*a*; electronic supplementary material, figure S2). Here, we focus on the lower postcanine dentitions as they show the largest range of variation and have been studied previously [26,27]. Whereas seal dentitions are relatively derived from the basic carnivoran pattern, seal postcanine morphologies are reminiscent of various pretribosphenic patterns in mammalian evolution, classified into different families and orders [28,29].

The overall morphologies of the grey and ringed seal postcanine teeth are markedly different. Ringed seal teeth have three to five slender cusps, and the teeth are generally more similar along the jaw (figure 2*a*; electronic supplementary material, figure S1*a* and table S6). By contrast, especially the anterior postcanine teeth of the grey seal have large, fang-shaped central cusps with small, variably present accessory cusps (figure 2*a*; electronic supplementary material, figure S1*a* and table S6). These morphological differences reflect the use of smaller fish and invertebrate foods by ringed seals compared with grey seals that, in addition to fish, are known to prey on mammals [30,31].

In the context of the other hybridizing species pairs (figure 1), the grey–ringed seal dental disparity is 1.74 cusps/tooth (difference in the number of cusps, see Material and methods), which is at least twice that of modern human–Neanderthal disparity (0.86 cusps/tooth, tabulation excluding accessory cusps is 0.54 cusps/tooth, figure 1; electronic supplementary material, table S7). The cusp number disparity is essentially zero in donkey–horse and lion–tiger pairs (figure 1), indicating that the seals represent the extreme phenotypic bracket for the species pairs studied.

Because teeth with large differences in cusp number are not readily analysed using standard morphometric methods, we quantified the hybrid morphology in relation to the two seal species by using a top-cusp angle, a measure of relative cusp height used previously in the analyses of seal dentitions (see Material and methods) [26,27]. The results show that the hybrid teeth are intermediate between a sample (*n* = 130) of the parent species by having relatively large central cusps and also relatively prominent accessory cusps (figure 2*a*; electronic supplementary material, figures S2 and S3). The distinct and intermediate morphology of the hybrid is most readily visible in the anterior postcanines (P2 and P3) in which the species differences are also the greatest due to the stronger anteroposterior gradation in the grey seal dentition (figure 2*a,b*; electronic supplementary material, figure S3). In addition, tooth size also appears intermediate between the grey and ringed seal (figure 2*a*). Because of the intermediate morphology of the hybrid, next we examined what kind of developmental changes might drive the observed patterns.

### 2.3. Developmental basis of the hybrid teeth

Genetic regulation of mammalian tooth development is highly conserved [32], and reiterative activation of signalling centres, called secondary enamel knots, directs cusp development in all studied mammals [32]. Consequently, computational modelling of genetic interactions and tissue biomechanics, based on empirical data on mouse tooth development, has been used to model tooth development of rodent [33,34] and seal species [27]. As neither seal tooth development nor hybridization is amenable to experimentation, we examined whether the hybrid morphology could be produced by modelling development.

First, we generated virtual ringed and grey seal tooth rows (using real population mean shapes, see Material and methods) using the ToothMaker software [33,34] (see Material and methods). Setting three model parameters to different values was sufficient to model the differences between grey and ringed seal teeth; inhibitor (*Inh*), epithelial growth rate (*Egr*) and anterior bias (*Abi*). These parameters, by regulating the dynamics of development, affect the spacing of cusps, the pointedness of cusps and the anterior–posterior symmetry of teeth, respectively (electronic supplementary material, figure S4*a*; see Material and methods). Changes along the tooth row were produced by a constant change in *Egr* in ringed seal models and a constant change in *Egr* and *Inh* in grey seal models (electronic supplementary material, table S8; see Material and methods). The constant parameter changes parsimoniously account for well-recognized gradual shape changes along the tooth row [35–38].

To mimic the hybridization between grey and ringed seals, the development of each tooth along the jaw was simulated after adjusting each of the three parameters separating the two species. For each parameter, we used either the average parameter value (assuming no dominance) or value adjusted 10% towards each of the parents. Additionally, we simulated teeth by keeping one or two of the parameters at the parent values. We then simulated tooth development using all these parameter value combinations (see Material and methods; electronic supplementary material, figure S4*b* and tables S8–S10).

The resulting simulated tooth shapes show that the hybrid cusp patterns can be produced by averaging the three parameters between the modelled grey and ringed seals (figure 2*c*). Furthermore, the top-cusp angles of this simulated hybrid tooth row fall between the parent shapes similarly to the real hybrid (figure 2*d*; electronic supplementary material, figure S4*b* and tables S9 and S10), suggesting that the regulatory principles of tooth shape are largely conserved between the two seal species.

A key parameter differentiating the grey and ringed seal teeth that is also required to be closest to the average between the parents is the inhibitor (*Inh*)*,* as otherwise the simulated hybrid would have the top-cusp angle close to one of the parents (figure 2*d*; electronic supplementary material, tables S9 and S10, and figure S4*b*). Experiments on developing mouse teeth have identified sonic hedgehog (SHH) as one candidate molecule for inhibition of new enamel knots and cusps [33]. However, because cusp spacing can be altered by tinkering with the activator–inhibitor balance of enamel knots through several molecules, these results do not necessarily implicate a single gene underlying each parameter. Rather, regardless of whether tooth shape is driven by a large number of loci or relatively few loci with major phenotypic effects (for discussion, see [17]), our simulation results are strongly suggestive that the hybrid dentition is both phenotypically and developmentally a predictable intermediate between the species.

### 2.4. Analysing the cranium of the hybrid

Unlike the dentition, the cranium of the newborn hybrid does not allow direct comparison to adult seals. However, differences in cranium morphology can be compared using geometric morphometrics across species and age groups [39], and we digitized 46 three-dimensional landmarks from newborn to adult grey and ringed seals (electronic supplementary material, figure S5 and table S11, *n* = 116; electronic supplementary material, movie S1; see Material and methods) to depict overall changes in skull shape.

**Figure 3.**
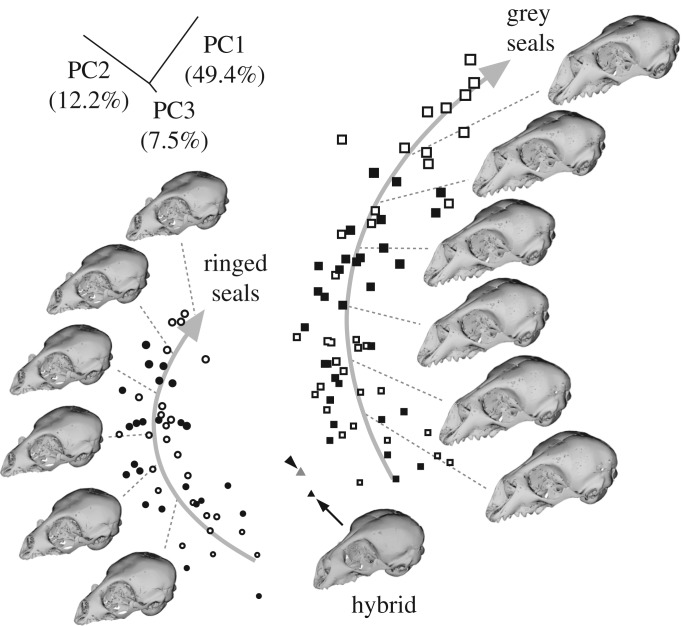
Ringed and grey seal crania are distinct at the birth and the hybrid is intermediate between the species. PCA shows the developmental trajectories from newborn to adult ringed (circles, *n* = 49) and grey (squares, *n* = 66) seals. Curved arrows indicate developmental trajectories obtained by multivariate quadratic regressions of shape onto centroid size. The symbol size represents the relative centroid size of the crania and open symbols are male, black female. Arrowhead denotes the geometric mean calculated using the youngest ringed (*n* = 11) and grey seal (*n* = 11) crania. See electronic supplementary material, figures S5 and S6; and see Material and methods.

The results show that grey and ringed seal crania have distinct developmental trajectories, and the newborn crania from each species are already well separated in the morphospace with no overlap (*p* < 0.0001, permutation test on Procrustes distance with 10 000 rounds, figure 3). The hybrid cranium is positioned between the two species and in the proximity of the geometric morphometric mean of newborn grey and ringed seals (figure 3). Therefore, as is the case for the dentition, the overall morphology of the cranium appears to be an intermediate between the species, a result agreeing with recent results on hybrids between different mouse strains [40].

Examining the details of cranial shape shows that the first two principal components (PCs) distinguish features that separate the two species (explaining 61.6% of total variance), while the third component captures some mixed traits common to both of them (explaining 7.5% of total variance) (electronic supplementary material, figure S6*a*). The hybrid differs from the geometric intermediate by having a shorter snout, narrower zygomatic arches and a more elongated braincase (electronic supplementary material, figure S6*b*,*c*), agreeing with the original description reporting that some details of the hybrid cranial morphology are closer to one or the other species [18]. We note, however, that the actual parents of the hybrid are not preserved, and thus, some details of the morphology could represent one of the individual parents. It is also possible that the third component describes unique differences of the hybrid from both of the parent species. Another factor affecting cranial shape is pronounced sexual dimorphism present in grey seals but not in ringed seals (figure 3), although we did not detect dimorphism in newborn crania (*p* = 0.485 for grey seals and *p* = 0.839 for ringed seals, permutation tests on Procrustes distances with 10 000 rounds). Overall, the intermediate cranial and dental features of the seal hybrid analysed suggest that a largely intermediate fossil specimen between two genera could potentially be a hybrid.

### 2.5. Detecting hybridization in the wild

Finally, because the grey–ringed seal hybrid was born in captivity, in itself it does not imply that comparable hybridizations happen in the wild. However, as is the case for mammals in general [4,6,16,40], several seal species are well established as hybridizing in the wild, including documentation of fertile intergeneric hybrids [41] and a living intergeneric hybrid between the dentally disparate harp seal (*Pagophilus groenlandicus*) and hooded seal (*Cystophora cristata*) [42]. Furthermore, behavioural observations have documented attempted matings by a wild grey seal male with harbour seal females (*Phoca vitulina*) [43].

**Figure 4.**
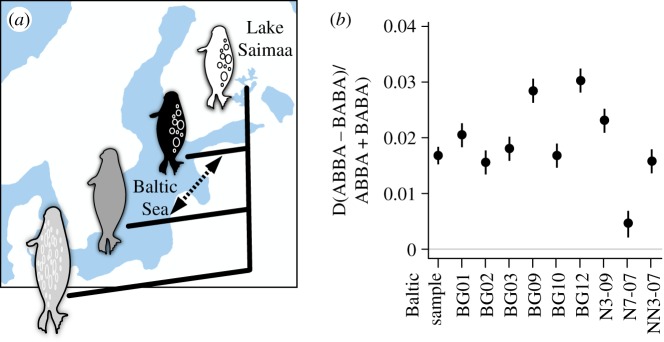
Introgression analysis suggests interbreeding between Baltic grey and ringed seals in the wild. (*a*) Saimaa ringed seals (white) have been landlocked in the Lake Saimaa, Finland for 9500 years, providing a suitable comparison for Baltic ringed seals (black) and grey seals (grey). Outgroup was the Weddell seal (light grey). (*b*) Positive *D*-statistic values, calculated using *D* (Saimaa ringed seal, Baltic ringed seal; Baltic grey seal, Weddell seal), indicate gene flow between Baltic grey and ringed seals (arrow in *a*). Although the *Z*-scores vary between the individuals, all but one individual (N7-07) show significant introgression (electronic supplementary material, table S12 and see Material and methods). Whiskers denote one standard error.

To estimate potential interbreeding in the history of wild grey and ringed seals, we examined introgression using the Patterson's *D*-statistics approach [8]. In addition to Baltic grey seals and Baltic ringed seals, we sequenced genome-wide data from Saimaa ringed seals (*n* = 12, see Material and methods). Saimaa ringed seals are a suitable contrast for the analyses (figure 4*a*), because they have been isolated from the Baltic for approximately 9500 years, with no documented presence of grey seals in Lake Saimaa [44,45]. The results show that Baltic ringed seals have a statistically significant excess of derived alleles shared with the grey seal compared to the Saimaa ringed seals (figure 4*b*; electronic supplementary material, table S12). We consider these results to be at least suggestive of interbreeding occurring in the Baltic between grey and ringed seals, and it is plausible that many seal populations and species will exhibit gene flow comparable to that in bears [46] and horses [25]. Taken together, the captive grey–ringed seal specimen can be considered to be a representative example of a phenotypically disparate hybridization.

## 3. Conclusion

Grey and ringed seals have sufficiently morphologically different dentitions that, if they were to be discovered as unknown fossils, at least a genus-level distinction could easily be justified. Even though intergeneric hybrids are well known to occur in many vertebrate groups [41,47,48], mammals have been suggested to have more limited capacity for hybridization due to faster evolutionary rates [49]. Yet, the combination of our genomic, phenomic and developmental analyses shows that grey and ringed seals can, and are likely to hybridize and that the resulting phenotype is a predictable combination of the two species. In more general terms, this hybrid bracket is indicative of the conservation of developmental mechanisms that tinker with quantitative traits. Whereas the overall conservation of developmental signalling in tooth development across the whole of jawed vertebrates is well established [32,50], our results suggest interspecies and higher-level conservation of the regulatory architecture underlying dental form. Interestingly, previous reports have found increased occurrences of supernumerary teeth in mammalian hybrids [1,51], which could indicate that there is a point of divergence at which the developmental regulation begins to lose canalization. Supernumerary teeth are relatively common in seal populations [52,53], but these are usually attributed to relaxed selection due to the lack of refined occlusion in seals [32,52,53]. Analyses of covariation between occluding teeth [54] together with genetic data may help to determine the potential roles of relaxed functional constraints and interbreeding in the occurrence of seal supernumerary teeth. Similarly, detailed analyses of the location and function of genomic regions showing introgression will offer a more nuanced view of the introgression reported here.

In the case of paleontological research where phenotypic analyses are the basis for taxonomic inferences, our results suggest that closely related genera could potentially hybridize, and that intermediates between distinct species may be hybrids themselves. The large morphological differences relative to the relatively modest genetic distance between grey and ringed seals are suggestive of an adaptive radiation phase of evolution, and we postulate that phenotypically disparate hybridizations are most probably to be observed in such radiations. In itself, hybridization between species has been also proposed to facilitate adaptive radiations [40,55]. Finally, in our case, the hybrid seal was of the first generation, and continuing interbreeding would result in segregation of traits, something that might appear as a mosaic mixture of traits in the fossil record [40]. Because both the genetic and dental distances of grey–ringed seals are more than twice those of Neanderthal–modern human distances, they bracket the overall hybridization potential in human ancestry and lead to an expectation of additional cases of hybrids in human ancestry [56].

## 4. Material and methods

### 4.1. Specimen preparation

The permanent teeth of the newborn hybrid were erupting and partially mineralized. Many of the teeth had cracked into halves during storage. The specimen was microCT scanned using a custom-built microtomography system (Nanotom 180 NF, General Electric, Wunstorf, Germany) at the Department of Physics, University of Helsinki, Finland. The voxel size was 32.1 µm for the skull and 16.6 µm for the teeth. Volume data were processed in Avizo 9.0 (FEI Visualization Sciences Group) and teeth were segmented from the jaw using Artec Studio 9.0 (Artec 3D). Cracked tooth halves were manually reattached using Artec Studio 9.0 without deforming the meshes (electronic supplementary material, figure S2). Skull images are from specimens in the Finnish Museum of Natural History (Helsinki, Finland) and partly for *Homo* from Tattersall & Schwartz [57].

### 4.2. Seal genetic data

Hybrid DNA was isolated from the pulp of a tooth that had fallen from the specimen in storage using the NucleoMag kit (Macherey-Nagel GmbH & Co., Germany). The same method was used to isolate DNA from Baltic grey seal, Baltic ringed and Saimaa ringed seal muscle tissue samples. All libraries and sequencing were performed at the DNA Sequencing and Genomics Laboratory, Institute of Biotechnology, University of Helsinki, Finland. The hybrid seal genome was sequenced with two separate runs of Illumina HiScanSQ platform and 11 separate runs of Illumina MiSeq platform to approximately 100× raw sequencing coverage (electronic supplementary material, table S1). The base calls in the Illumina HiScanSQ platform were converted into text format (FASTA-format with individual base quality scores) using the CASAVA toolkit (bcl2fastq v. 1.8.3) provided by the manufacturer. The Illumina MiSeq platform employed a primary analysis software MiSeq Reporter post-run that produced the base calls in text format.

For all the Illumina reads, base call accuracy and read length filtering (adapter cut-off) were performed with cutadapt [58] using minimum accepted base call accuracy of 90% and minimum post-filtering read length of 75 bp. The Weddell seal (*Leptonychotes weddellii*) draft genome (Broad Institute) was used as a reference genome for the downstream genetic distance analysis.

### 4.3. Admixture and introgression analyses

Short read data from seals were mapped to the Weddell seal reference genome using bwa mem (v. 0.7.15), samtools rmdup (v. 1.3.1) and GATK IndelRealigner (v. 3.7). The resulting bam files were analysed using ANGSD [59]. The admixture analysis of the hybrid was carried using NgsAdmix [60] on a random sample of 3.6 million markers (10% of the data). NgsAdmix was run 10 times with *K* = 2 clusters and all the runs converged to identical clustering. Introgression was studied using Patterson's *D*-statistics [8] that, other than requiring the ancestral population to be randomly mating, is robust to variables such as variations in population size [61]. The statistic was calculated with the ANGSD's abbababa2 module for the first 1000 scaffolds (over 50% of the genome) having the Saimaa and Baltic ringed seals as P1 and P2, and Baltic grey seals as P3. To examine individual differences, the *D*-statistic was calculated for each Baltic individual.

### 4.4. Genetic distances

We analysed fourfold degenerate (ffd) sites from a large number of single-copy orthologous genes. Human genes and their orthologues in cat, dog, horse and chimpanzee were fetched from Ensembl BioMart (v. 83) [62], resulting in 4826 complete gene sets with unambiguous orthology (‘Homology Type’ is ‘ortholog_one2one’; ‘Orthology confidence’ is ‘high’). Nucleotide and peptide sequences of transcripts were fetched using the Ensembl REST API [63]. Using last (v. 658) [64], scaffold-level genome assemblies of tiger, Weddell seal and donkey were aligned against the genomes of cat, dog and horse, respectively. Lift-over chains were built using the Kent source utilities [65]. Short read data for donkey and lion were mapped to the horse and tiger genomes using bwa mem (v. 0.7.15), samtools rmdup (v. 1.3.1) and GATK IndelRealigner (v. 3.7) [66,67]. Locally generated short read data for Baltic grey–ringed seals were mapped to the Weddell seal genome. The data for the Neanderthal human individual were obtained as bam alignment files. Data sources are listed in electronic supplementary material, table S2.

The annotations were divided into individual coding exons, removing split codons where necessary, and the exon coordinates in reference species (human, cat, dog and horse) were transferred to related non-reference genomes using CrossMap (v. 0.2.4) [68]. For each transcript, exons were extracted from the non-reference genomes using samtools (v. 1.3.1) and matched against the reference peptide using Pagan's (v. 0.61) translated pileup alignment [69]. The resulting back-translated nucleotide alignments were flattened and degapped, combining all successfully extracted exons into one sequence. For species with bam alignment data (donkey, grey and ringed seal, Neanderthal), the regions surrounding the exons were reconstructed by placing observed sequence changes to the genome sequences of horse, Weddell seal and human. For each exon separately, variants were inferred with samtools mpileup and bcftools call (v. 1.3.1) and then placed to the genome sequences with vcfutils vcf2fq, requiring sequencing depth of 10 or higher. The exon regions were extracted and combined into one sequence.

Resulting sequences were aligned and the longest transcripts were selected using Pagan's translated pileup alignment. Multiple sequence alignments were filtered and regions where two neighbouring codons contain more than one non-identical base among the species were masked. For different species sets, gene sequence alignments of at least 100 columns in length after filtering and removal of sites with missing data were concatenated, and ffd sites were extracted using R package rphast (v. 1.6.5) [70]. Pairwise genetic distances were computed using R package ape (v. 4.0) [71] and model T92, and phylogenetic trees inferred with RAxML (v. 8.2.9) [72] using model GTRGAMMA. In addition, we measured the distances using all the codon third sites, and the relative distances remained largely the same. The different species sets analysed are in electronic supplementary material, tables S3–S5.

### 4.5. Dental material and analyses

Baltic grey and ringed seal material was used in comparison to the hybrid as the parents were reported to be from the Baltic Sea (electronic supplementary material, dataset S1 and table S6). The number of cusps and top-cusp angles were tabulated from images taken from lingual side using ImageJ (v. 1.51). The angle captures the relative height of the tallest cusp and correlates with cusp number because cusp spacing and number are developmentally linked [26,27]. For teeth that had only one or two cusps, the line was drawn to the maximum anterior or distal ends at the crown base. Our tabulations included small incipient cusps (arrowheads in electronic supplementary material, figure S2*b*). All the measurements reported are from the right side. The pattern of results remains the same for the left side. We excluded jaws with anomalies such as extra teeth, and individual teeth with cracked or worn cusp tips (65 non-anomalous jaws were randomly chosen for each species). A sample of grey and ringed seal teeth were imaged and measured twice to examine the robustness of the angle measurements (mean absolute error = 1.0 and 1.5, s.d. = 0.92 and 1.08, *n* = 40 and 46 teeth for grey and ringed seals, respectively). To test how intermediate the hybrid is between the species, we calculated average top-cusp angles between each grey–ringed seal pair and tabulated the probability of obtaining the hybrid value from the species averages (figure 2*b*; electronic supplementary material, figure S3). For visualization, dental specimens were scanned with PlanScan laser scanner (PlanMeca, Helsinki, Finland).

To measure disparity between dentitions [73], we tabulated the absolute difference in cusp number for all lower postcanine teeth as this measure captures also fundamental differences in tooth patterning. For seals, we used the cusp numbers of teeth P1 to P5 (electronic supplementary material, table S7). For humans, we used the Neanderthal and fossil modern human tabulations by Martinón-Torres *et al.* [74] with some modifications. For premolar scoring, grades 1 and 2 were scored as one cusp, and grade 5 as four cusps. Molar hypoconulid was tabulated to be present for grade 2 or higher. We also tabulated the molar accessory cusps C6 and C7 to be present for grades 2 or higher. We did not tabulate the lingual accessory cusps sometimes present in Baltic ringed seals, and the seal disparity values should be considered as minimum estimates. The disparity values were calculated using the mean and mode cusp number for each tooth separately due to the limited availability of whole jaw data. Lion and tiger disparities were here marked to be zero as these dentitions are almost indistinguishable at the level of cusps. Even if lower P4 might occasionally have a fourth cusp in the talonid, this, or the distal notch of M1, would not affect the pattern of the results. Horses have hypsodont molars with essentially invariant number of cusps. Even though it is possible to distinguish donkey and horse postcanines based on subtle enamel folding patterns [75], these differences in cusp shape rise after the patterning of cusp number.

### 4.6. Developmental modelling of teeth

We used ToothMaker [33] to simulate seal tooth development. The model integrates experimentally inferred genetic interactions with tissue biomechanics to simulate tooth development [27,33,34]. We focused on three parameters to simulate species differences. To regulate spacing of cusps, the key difference between grey and ringed seals, we adjusted the strength of the enamel knot-secreted inhibition of enamel knot formation (parameter *Inh*). The other two parameters are epithelial growth (*Egr*) and anterior bias (*Abi*). *Egr* affects the relative growth of the epithelium and pointedness of cusps, and *Abi* the anterior extension of the tooth germ; both have been found previously to account for variation in species differences [27,33,34]. Because seals have laterally compressed teeth, lateral biases (*Lbi* and *Bbi*) were kept constant [27]. All the used parameter values are listed in the electronic supplementary material, table S7. We modelled tooth rows by making constant parameter changes from tooth to tooth to mimic gradual shape changes along the jaw. All the simulations were run for the same number of iterations. Hybridization was simulated by averaging parameter values between the species in different combinations (electronic supplementary material, figure S4*b* and table S8). ToothMaker is available at https://github.com/jernvall-lab/ToothMaker.

### 4.7. Analysis of the crania

Forty-six three-dimensional landmarks obtained from ventral and dorsal views were acquired from newborn to adult skulls of grey and ringed seals from the collections of Finnish Museum of Natural History (Helsinki, Finland) using a Microscribe G2X (Immersion) (electronic supplementary material, figure S5), with digitizing error also assessed (electronic supplementary material, table S11). A generalized Procrustes analysis was used to simultaneously superimpose all symmetrized configurations and extract shape data by removing the effects of scale, orientation and position [39]. Permutation tests were used for testing differences in mean shapes using function ‘permudist’ from ‘Morpho’ R package [76].

A principal component analysis (PCA) was used to show the main patterns of morphological variation between the two species (electronic supplementary material, dataset S2). A three-dimensional microCT scan of the hybrid was warped to the consensus using the thin plate spline method via the function ‘warpRefMesh’ in geomorph [77] and the main patterns of shape changes were visualized as deviations from this warped consensus for the first three PCs (electronic supplementary material, figure S6*a*). Differences between the hybrid and the two species were visualized via warping and heatmaps (electronic supplementary material, figure S6*b*,*c*). A multivariate quadratic regression of shape onto centroid size was used to investigate the main patterns of allometric growth for each species that were visualized with warped three-dimensional microCT scans of a newborn grey and ringed seal using Landmark Editor 3.6 and function ‘warpmovie3d’ (figure 3; electronic supplementary material, movie S1) [76,78]. All analyses were performed with MorphoJ 1.06d [79], custom R [80] and Python scripts.

## Supplementary Material

Supplementary figures, tables and datasets

## Supplementary Material

Supplementary movie

## Data accessibility

The aligned sequences are available through the European Nucleotide Archive under accession number PRJEB25679 (https://www.ebi.ac.uk/ena/data/view/PRJEB25679) with additional analysis data at http://wasabiapp.org/download/data/bastard/. Software is available from https://github.com/jernvall-lab/ToothMaker. All other data used in the analyses are available in the electronic supplementary material. The cranial and dental specimens are archived in the Finnish Museum of Natural History (Helsinki) and Swedish Museum of Natural History (Stockholm).
