# Supplementary material for: Bracketing phenogenotypic limits of mammalian hybridization
Source: R Soc Open Sci. 2018 Nov 28;5(11):180903. doi: 10.1098/rsos.180903 (PMC6281900; doi:10.1098/rsos.180903)
Supplement: Supplementary figures, tables and datasets [file rsos180903supp1.docx]

**Supplementary Materials**

*Royal Society Open Science*

**Bracketing phenogenotypic limits of mammalian hybridization**

Yoland Savriama^1^†, Mia Valtonen^1,2^†, Juhana I. Kammonen^3^†, Pasi Rastas^3^, Olli-Pekka Smolander^3^, Annina Lyyski^3^, Teemu J. Häkkinen^1^, Ian J. Corfe^1^, Sylvain Gerber^4^, Isaac Salazar-Ciudad^1,5^, Lars Paulin^3^, Liisa Holm^3,6^, Ari Löytynoja^3^*, Petri Auvinen^3^*, Jukka Jernvall^1^*

^1^Developmental Biology Program, Institute of Biotechnology, University of Helsinki, P.O. Box 56, FIN-00014 Helsinki, Finland.

^2^Department of Environmental and Biological Sciences, University of Eastern Finland, P.O. Box 111, FIN-80101 Joensuu, Finland.

^3^Genome Biology Program, Institute of Biotechnology, University of Helsinki, P.O. Box 56, FIN-00014 Helsinki, Finland.

^4^Institut Systématique Evolution Biodiversité (ISYEB), Muséum national d'Histoire naturelle, CNRS, Sorbonne Université, EPHE, 45 rue Buffon, CP 50, 75005 Paris, France.

^5^Departament de Genètica i Microbiologia, Universitat Autònoma de Barcelona, 08193 Cerdanyola del Vallès, Spain.

^6^Faculty of Biological and Environmental Sciences, University of Helsinki, P.O. Box 56, FIN-00014 Helsinki, Finland.

†These authors contributed equally to this work.

*For correspondence: ari.loytynoja@helsinki.fi, petri.auvinen@helsinki.fi, or jernvall@fastmail.fm.

**Six figures:** figure S1 to S6

**Twelve tables:** table S1 to S12

**Two datasets:** dataset S1 and S2

**Movie S1 caption**

**
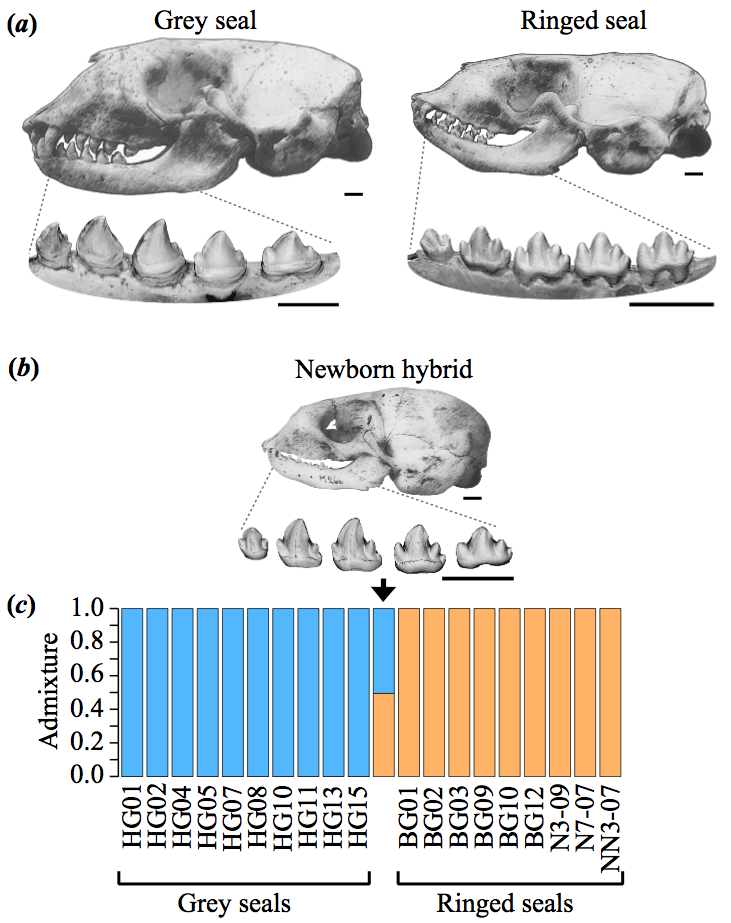
**

**Figure S1.** Grey and ringed seal cranio-dental morphologies show at least genus-level differences and genetic data validates the hybrid specimen. (*a*) The grey seal skull has a prominent snout with a straight profile whereas the overall cranial morphology of ringed seals is relatively gracile. The grey seal lower postcanines have robust, fang-shaped central cusps with small and variably present accessory cusps. There is a marked gradation of tooth shapes along the jaw, and grey seal teeth are approximately 40% larger (anteroposteriorly) than ringed seal teeth. The ringed seal lower postcanines have typically four slender cusps on the four large postcanines of the lower jaw. Upper teeth show comparable but less disparate differences in cusp number. Grey seals have pronounced sexual dimorphism and the skull illustrations are of females. Postcanine dentitions, showing obliquely lingual views, have no marked sexual dimorphism. (*b*) The hybrid specimen is a newborn skull and mandible with erupting, but fully formed teeth. (*c*) Genetic analysis from hybrid dental pulp shows close to 50% admixture between grey (50.51%) and ringed (49.49%) seals. Analysis shown (K=2) was performed using NGSadmix of ANGSD with 3.6 million markers (see Materials and Methods). Scale bars, 10 mm.


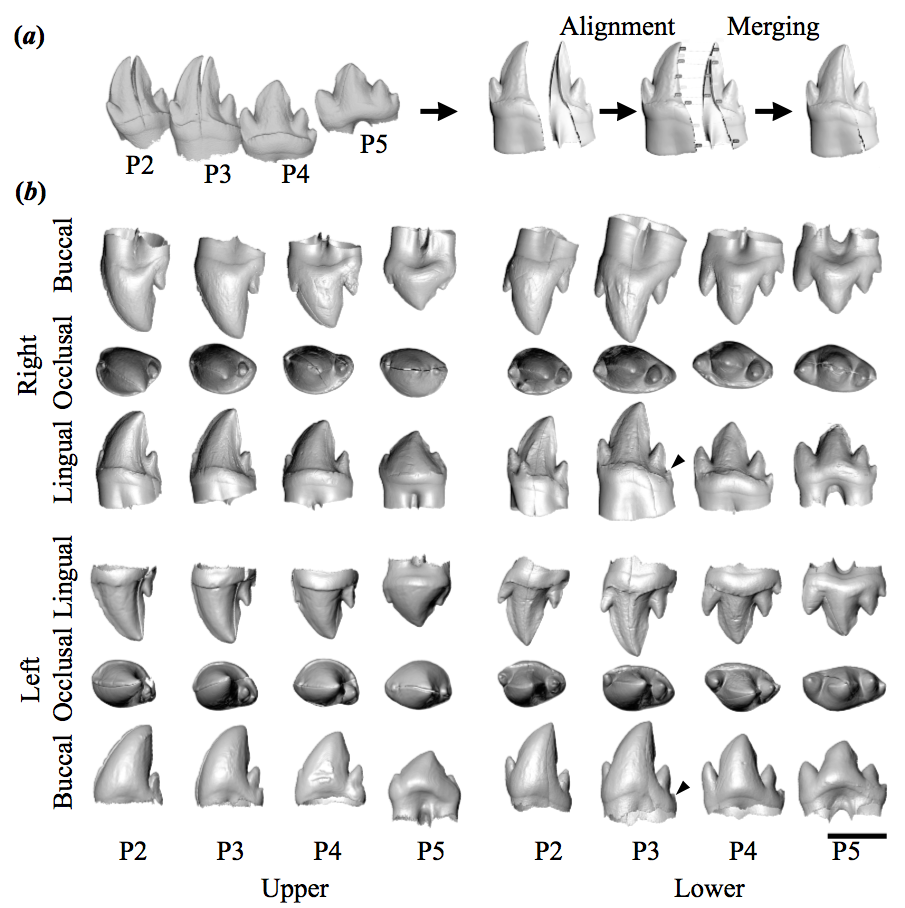


**Figure S2.** Reconstructions of the hybrid postcanine dentitions. (*a*) The teeth of the hybrid were imaged using microCT and three-dimensional reconstructions of the cracked teeth were aligned and merged. (*b*) Reconstructed hybrid dentitions. Postcanines P2 to P4 are replacement teeth to vestigial deciduous teeth, whereas P5 is the first permanent molar. Arrowheads in lower dentition show incipient cusps that were included in the cusp number tabulations. Lower right P2 has a small extra lingual cusp. Lingual accessory cusps are variably present in Baltic ringed seals. Scale bar, 5 mm.


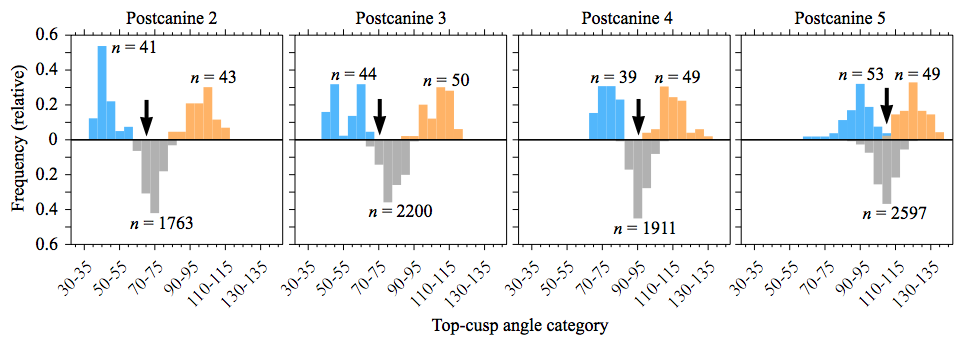


Figure S3. Top-cusp angle distributions are distinct between the species and the hybrid is intermediate. Grey seal (blue colour) and ringed seal (orange colour) top-cusp angles are distinct, but progressively more similar towards the posterior teeth. The mirrored histograms (grey colour) show the distribution of top-cusp angles averaged between each grey-ringed seal pair. The arrows mark the top-cusp angles of the hybrid (within the five-degree bins). Probabilities, calculated as one-tailed frequencies of observing the hybrid values, or lower for P2 to P4 and higher for P5, among the averaged shapes are *p* = 0.324, 0.090, 0.461, and 0.312, for P2, P3, P4, and P5, respectively (calculated from unbinned data). The bimodal distribution of grey seal P3 is due to the variable presence of accessory cusps affecting the top-cusp angle (mean angles for one, two, and three cusped P3s are 46.2, 61.1, and 61.7 degrees, respectively). Although the actual parents of the hybrid have not been preserved, it is plausible that the P3 of the father lacked accessory cusps since the hybrid is an intermediate between the ringed seals and unicusped grey seals.


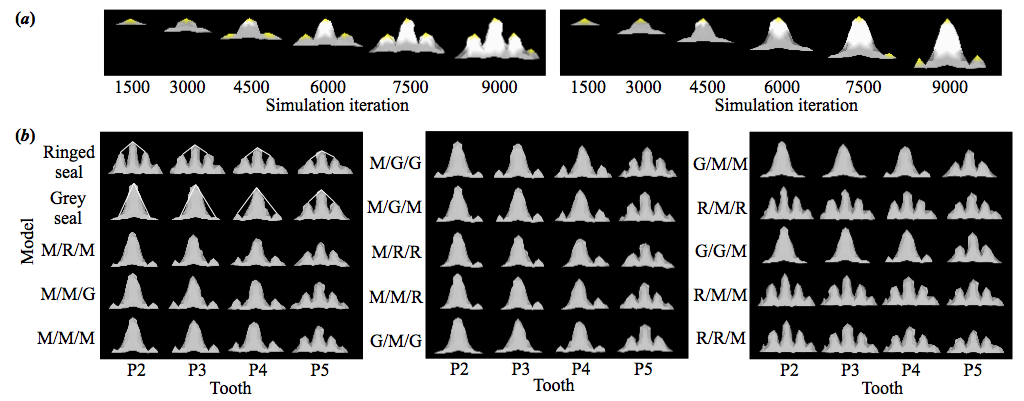


Figure S4. Computational simulation of seal tooth development. (*a*) ToothMaker simulates tooth development and visualizes the shape of the inner enamel epithelium that will grow until it differentiates (white). Epithelial signalling centres, enamel knots (yellow), cause local cessation of growth but they also promote the growth of the surrounding tissue and inhibit the formation of new enamel knots by secreting inhibitors. Simulation of seal tooth development with weak inhibition (on the left) allows enamel knots and cusps to appear quickly, whereas strong inhibition (on the right) causes enamel knots and cusps to appear slowly. (*b*) Different simulations of the hybridisation in which mean (M), grey seal (G), or ringed seal (R) values were used for the *Inh*, *Egr*, and *Abi* parameters, respectively. The hybridisations are in the increasing order that the average top-cusp angle of the tooth row differs from the real hybrid (Table S10). The simulated grey and ringed seals show how the top-cusp angles were measured.

Figure S5. 3D landmarks used for geometric morphometrics of the cranium. Landmarks were acquired on ventral (*n* = 31) and dorsal (*n* = 22) views of skulls. Landmarks labelled in yellow colour were used to merge the configurations into a single one, and the total number of landmarks used was 46.

Figure S6. Main patterns of cranial morphological variation along the first three PCs. Visualised using 3D-warped surface scans obtained via Thin-Plate Spline (TPS) and comparison between the hybrid and the geometric intermediate. (*a*) Shape changes associated with each PC from the consensus shape for negative and positive Procrustes distances of 0.06, for five orthogonal views (the same PCA as in Fig. 4). (*b*) Morphological comparison of the hybrid (solid 3D surface) to the geometric intermediate (translucent 3D surface). The differences are amplified five times for better visibility. (*c*) Heatmaps pinpointing the direction and localization of morphological congruence between the hybrid (solid 3D surface) and the geometric intermediate.

**Table S1.** Genetic data of the hybrid.

| Platform | Read length (bp) | Read pairs | Orphan reads | Total reads | Total reads passed Q≥10* |
| --- | --- | --- | --- | --- | --- |
| HiScanSQ | 100 | 464,666,847 | 106,161,451 | 1,170,310,720 | 1,033,529,704 |
| MiSeq | 250-300 | 110,287,755 | 157,596 | 189,837,698 | 189,603,446 |

*Phred score q≥10 equals base call accuracy of at least 90%.

Table S2. Genetic data used for distance calculations.

| **Species** | **Version/File/ID** | **Source** |
| --- | --- | --- |
| Grey seal | Hg-01 | ERS2313726 |
| Ringed seal | BG-03 | ERS2313698 |
| Lion | SRR836361 | https://www.ncbi.nlm.nih.gov/sra |
| Tiger | scaffold_merged | ftp://biodisk.org/SiberianTiger/Scaffold |
| Donkey | SRR873443-5 | https://www.ncbi.nlm.nih.gov/sra |
| Horse | EquCab2 | http://www.ensembl.org |
| Human | hg19 | http://hgdownload.cse.ucsc.edu/goldenPath/hg19/chromosomes |
| Neanderthal | AltaiNea | http://cdna.eva.mpg.de/neandertal/altai/AltaiNeandertal |
| Weddell seal | L_weddellii_v0 | ftp://ftp.broadinstitute.org/pub/assemblies/mammals/manatee/LepWed1.0 |
| Cat | Felis_catus_6.2 | http://www.ensembl.org |
| Dog | CanFam3.1 | http://www.ensembl.org |
| Chimpanzee | CHIMP2.1 | http://www.ensembl.org |

**Table S3.** Genetic distances between hybridising species (substitutions per fourfold degenerate site).

|  | Human | Neanderthal | Tiger | Lion | Horse | Donkey | Ringed seal | |
| --- | --- | --- | --- | --- | --- | --- | --- | --- |
| Neanderthal | 0.0012 |  |  |  |  |  | |  |
| Tiger | 0.2596 | 0.2596 |  |  |  |  | |  |
| Lion | 0.2599 | 0.2598 | 0.0063 |  |  |  | |  |
| Horse | 0.2369 | 0.2368 | 0.2164 | 0.2165 |  |  | |  |
| Donkey | 0.2366 | 0.2365 | 0.2159 | 0.2160 | 0.0118 |  | |  |
| Ringed seal | 0.2539 | 0.2539 | 0.1541 | 0.1542 | 0.2084 | 0.2079 | |  |
| Grey seal | 0.2540 | 0.2539 | 0.1544 | 0.1544 | 0.2086 | 0.2082 | | 0.0031 |

Based on 1,526,260 codons, 623,391 fourfold degenerate sites, and 4,045 orthologous genes.

Table S4. Genetic distances between hybridising and other species (substitutions per fourfold degenerate site).

|  | Hum | Nea | Chi | Tig | Lio | Cat | Hor | Don | Rin | Gre | Wed |
| --- | --- | --- | --- | --- | --- | --- | --- | --- | --- | --- | --- |
| Nea | 0.0012 |  |  |  |  |  |  |  |  |  |  |
| Chi | 0.0108 | 0.0108 |  |  |  |  |  |  |  |  |  |
| Tig | 0.2554 | 0.2554 | 0.2554 |  |  |  |  |  |  |  |  |
| Lio | 0.2556 | 0.2556 | 0.2556 | 0.0062 |  |  |  |  |  |  |  |
| Cat | 0.2554 | 0.2554 | 0.2554 | 0.0189 | 0.0186 |  |  |  |  |  |  |
| Hor | 0.2335 | 0.2334 | 0.2334 | 0.2136 | 0.2137 | 0.2135 |  |  |  |  |  |
| Don | 0.2331 | 0.2330 | 0.2331 | 0.2132 | 0.2132 | 0.2130 | 0.0117 |  |  |  |  |
| Rin | 0.2496 | 0.2496 | 0.2496 | 0.1519 | 0.1520 | 0.1520 | 0.2058 | 0.2053 |  |  |  |
| Gre | 0.2497 | 0.2497 | 0.2497 | 0.1522 | 0.1523 | 0.1523 | 0.2061 | 0.2056 | 0.0030 |  |  |
| Wed | 0.2501 | 0.2501 | 0.2501 | 0.1533 | 0.1534 | 0.1535 | 0.2071 | 0.2067 | 0.0193 | 0.0199 |  |
| Dog | 0.2615 | 0.2615 | 0.2614 | 0.1711 | 0.1711 | 0.1714 | 0.2221 | 0.2213 | 0.1359 | 0.1362 | 0.137 |

Based on 1,433,239 codons, 580,030 fourfold degenerate sites, and 3,955 orthologous genes. Hum = Human, Nea = Neanderthal, Chi = Chimpanzee, Tig = Tiger, Lio = Lion, Cat = Domestic cat, Hor = Horse, Don = Donkey, Rin = Ringed seal, Gre = Grey seal, Wed = Weddell seal, Dog = Dog.

**Table S5.** Genetic distances between hybridising species subsets (substitutions per fourfold degenerate site).

|  | Hum | Nea | Rin |  | Tig | Lio | Rin |  | Hor | Don | Rin |
| --- | --- | --- | --- | --- | --- | --- | --- | --- | --- | --- | --- |
| Nea | 0.0013 |  |  | Lio | 0.0067 |  |  | Don | 0.0125 |  |  |
| Rin | 0.2655 | 0.2655 |  | Rin | 0.1638 | 0.1639 |  | Rin | 0.2209 | 0.2204 |  |
| Gre | 0.2656 | 0.2656 | 0.0032 | Gre | 0.1641 | 0.1641 | 0.0032 | Gre | 0.2212 | 0.2208 | 0.0033 |

Based on 1,912,346 codons, 806,827 fourfold degenerate sites, and 4,166 orthologous genes for hominin-seal comparisons, 1,868,861 codons, 806,530 fourfold degenerate sites, and 4,231 orthologous genes for carnivore-seal comparisons, and 1,881,735 codons, 803,933 fourfold degenerate sites, and 4,216 orthologous genes for perissodactyl-seal comparisons. Hum = Human, Nea = Neanderthal, Tig = Tiger, Lio = Lion, Hor = Horse, Don = Donkey, Rin = Ringed seal, Gre = Grey seal.

Table S6. Measurements of grey and ringed seal dentitions.

|  |  | Cusp number | | | |  | Top-cusp angle | | | |
| --- | --- | --- | --- | --- | --- | --- | --- | --- | --- | --- |
| Species | Tooth | *n* | Mean | Range | SD |  | *n* | Mean | Range | SD |
| *Halichoerus* | P1 | 32 | 1.7 | 1–3 | 0.83 |  | - | - | - | - |
|  | P2 | 65 | 1.2 | 1–3 | 0.46 |  | 41 | 44.2 | 37.4–58.7 | 5.26 |
|  | P3 | 65 | 1.8 | 1–3 | 0.85 |  | 44 | 53.8 | 42.3–66.8 | 8.11 |
|  | P4 | 65 | 2.9 | 2–3 | 0.36 |  | 39 | 75.5 | 67.9–84.1 | 4.48 |
|  | P5 | 65 | 2.9 | 1–5 | 0.63 |  | 53 | 90.7 | 62.3–107.0 | 8.77 |
| *Pusa* | P1 | 37 | 3.1 | 2–4 | 0.33 |  | - | - | - | - |
|  | P2 | 65 | 4.0 | 4–5 | 0.12 |  | 43 | 98.8 | 81.7–112.0 | 7.34 |
|  | P3 | 65 | 4.1 | 4–5 | 0.33 |  | 50 | 105.8 | 89.5–117.1 | 6.99 |
|  | P4 | 65 | 4.2 | 3–5 | 0.43 |  | 49 | 112.3 | 98.4–131.9 | 7.38 |
|  | P5 | 65 | 3.8 | 3–5 | 0.69 |  | 49 | 122.6 | 109.7–139.1 | 7.31 |

Data are from the right side, Cusps have been tabulated regardless how large they are and contain incipient cusps. Angle measurements were not taken from teeth with cracked or worn cusp tips. P1 to P5 refers to first to fifth postcanine teeth. Individual specimen measurements are in (dataset S1). P1 cusp tabulations were used only in the disparity calculations (table S7).

Table S7. Dental disparity values for lower postcanine teeth.

| Grey-ringed seal disparity | | |  | Modern human-Neanderthal disparity | | | | |
| --- | --- | --- | --- | --- | --- | --- | --- | --- |
| Tooth | Disparity  (mean) | Disparity  (mode) |  | Tooth | Disparity  (mean) | Disparity  (mean,  excl. C6,C7) | Disparity  (mode) | Disparity  (mode,  excl. C6,C7) |
| P1 | 1.4 | 2 |  | P3 | 0.4 | 0.4 | 0 | 0 |
| P2 | 2.8 | 3 |  | P4 | 1.4 | 1.4 | 2 | 2 |
| P3 | 2.3 | 3 |  | M1 | 0.5 | 0.0 | 1 | 0 |
| P4 | 1.3 | 1 |  | M2 | 1.2 | 0.6 | 2 | 1 |
| P5 | 0.9 | 1 |  | M3 | 0.8 | 0.3 | 1 | 0 |
| Total | 8.7 | 10 |  | Total | 4.3 | 2.7 | 6 | 3 |
| Total/tooth | 1.74 | 2.0 |  | Total/tooth | 0.86 | 0.54 | 1.2 | 0.6 |

Data for seals are from this study and for fossil modern humans and Neanderthals from [74]. These are differences calculated using mean and mode cusp numbers. The human sample sizes are roughly half that of the seal samples. The variably present cusps in human teeth were lingual cusps in premolars and the hypoconulid, C6, and C7 in molars, see Material and Methods. Cusp numbers are essentially invariant in the other two species pairs (lion-tiger and donkey-horse).

Table S8. Model parameters to simulate seal teeth and hybridisation.

|  | | *Inh* | | | | *Egr* | | | | *Abi* | | | |
| --- | --- | --- | --- | --- | --- | --- | --- | --- | --- | --- | --- | --- | --- |
|  | | P2 | P3 | P4 | P5 | P2 | P3 | P4 | P5 | P2 | P3 | P4 | P5 |
| *Halichoerus* | 35.5 | | 24.5 | 13.5 | 2.5 | 0.026 | 0.025 | 0.024 | 0.023 | 17 | 17 | 17 | 17 |
| *Pusa* | 1.5 | | 1.5 | 1.5 | 1.5 | 0.025 | 0.023 | 0.021 | 0.019 | 12 | 12 | 12 | 12 |
| Hybrid | 18.5 | | 13.0 | 7.5 | 2.0 | 0.0255 | 0.024 | 0.0225 | 0.021 | 14.5 | 14.5 | 14.5 | 14.5 |

Parameters and their values that are different between simulated grey and ringed seal teeth. The hybrid values are the averages between the simulated grey and ringed seal values. The other parameters have identical values in all ToothMaker simulations: Iteration = 9000, Mgr = 200.0, Rep = 1.0, Swi = 0.0, Adh = 0.001, Act = 0.22, Sec = 0.03, Da = 0.2, Di = 0.2, Ds = 0.2, Int = 0.19, Set = 0.95, Boy = 0.1, Dff = 0.0004, Bgr = 1.0, Pbi = 18.0, Lbi = 1.0, Bbi = 1.0, Rad = 2.0, Deg = 0.1, Dgr = 10000.0, Ntr = 0.00001, Bwi = 0.8, Ina = 0.0. For parameters, see Methods and refs. [27, 33, 34]. The model has different seal parameter values as defaults (it can be downloaded at https://github.com/jernvall-lab/ToothMaker).

Table S9. Measurements of empirical and simulated dentitions.

|  | Cusp number | | | | Top-cusp angle | | | |
| --- | --- | --- | --- | --- | --- | --- | --- | --- |
| Sample or simulation | P2 | P3 | P4 | P5 | P2 | P3 | P4 | P5 |
| *Halichoerus* mean | 1.2 | 1.8 | 2.9 | 2.9 | 44.2 | 53.8 | 75.5 | 90.7 |
| Model *Halichoerus* | 1 | 2* | 3 | 3 | 49.5 | 57.3 | 75.7 | 93.4 |
| *Pusa* mean | 4.0 | 4.1 | 4.2 | 3.8 | 98.8 | 105.8 | 112.3 | 122.6 |
| Model *Pusa* | 4 | 4 | 4 | 4 | 100.2 | 105.3 | 110.4 | 117.5 |
| Hybrid (NRM A619266) | 3 | 4* | 4 | 4 | 69.4 | 71.8 | 93.2 | 109.5 |
| Model hybrid (mean) | 3 | 3 | 4* | 4 | 65.2 | 73.9 | 87.1 | 103.6 |
| Model hybrid -10%–+10% | 2–3 | 3 | 3–4* | 4–5 | 52.3–68.7 | 71.0–78.0 | 80.8–89.6 | 101.7–105.0 |

Mean values of real teeth and simulated teeth. Asterisks mark cusp numbers that include one incipient cusp. The hybrid bracketing in the bottom row is done by adjusting each parameter between the hybrid mean towards the grey seal or ringed seal value by 10%. The maximum range was obtained using *Inh*, except for P4 minimum that was obtained using *Abi*.

Table S10. Modelled hybrids and their distances from the real hybrid.

| *Inh* | *Egr* | *Abi* | Mean angle difference | Mean cusp number difference |
| --- | --- | --- | --- | --- |
| Mean | Ringed seal | Mean | 2.56 | 0.5 |
| Mean | Mean | Grey seal | 3.44 | 0.5 |
| Mean | Mean | Mean | 4.60 | 0.25 |
| Mean | Grey seal | Grey seal | 5.19 | 0.5 |
| Mean | Grey seal | Mean | 6.73 | 0.5 |
| Mean | Ringed seal | Ringed seal | 9.69 | 1.0 |
| Mean | Mean | Ringed seal | 13.51 | 1.0 |
| Grey seal | Mean | Grey seal | 15.83 | 1.5 |
| Grey seal | Mean | Mean | 16.73 | 1.5 |
| Ringed seal | Mean | Ringed seal | 18.69 | 0.75 |
| Grey seal | Grey seal | Mean | 19.59 | 1.5 |
| Ringed seal | Mean | Mean | 22.27 | 1.25 |
| Ringed seal | Ringed seal | Mean | 25.96 | 1.25 |

Mean of absolute differences calculated for each tooth for all the different simulations. Simulated dentitions are shown in figure S4.

Table S11. 3D-digitizing error relative to individual differences.

| Data | Effect | Sum of squares | df | Mean square | F | *p* |
| --- | --- | --- | --- | --- | --- | --- |
| Shape | Individual | 1.191 | 8050 | 0.0001479 | 63.99 | <0.0001 |
|  | Digitising error | 0.035 | 15196 | 0.0000023 |  |  |
|  | Individual | 1376145.000 | 115 | 11966.478 | 1068.90 | <0.0001 |
| Centroid Size | Digitising error | 1298.634 | 116 | 11.195 |  |  |

One-way ANOVAs show that digitizing error is small relative to variation among individuals.

Table S12. Introgression Z scores for individual seals and the sample.

|  | Baltic sample | BG-01 | BG-02 | BG-03 | BG-09 | BG-10 | BG-12 | N309 | N707 | NN3-07 |
| --- | --- | --- | --- | --- | --- | --- | --- | --- | --- | --- |
| Z score | 11.11 | 9.91 | 7.42 | 8.96 | 14.39 | 8.42 | 15.31 | 11.3 | 2.11 | 7.71 |
| *p* value | 0.0000 | 0.000 | 0.0000 | 0.0000 | 0.0000 | 0.0000 | 0.0000 | 0.0000 | 0.0353 | 0.0000 |

P values testing against the null hypothesis of lack of introgression is evaluated by weighted block jackknife in ANGSD. The European Nucleotide accession numbers for all the samples are ERS2313526, ERS2313598, ERS2313603, ERS2313604, ERS2313605, ERS2313606, ERS2313607, ERS2313608, ERS2313610, ERS2313611, ERS2313616, ERS2313691, ERS2313692, ERS2313693, ERS2313697, ERS2313698, ERS2313699, ERS2313700, ERS2313701, ERS2313702, ERS2313724, ERS2313725, ERS2313726, ERS2313727, ERS2313756, ERS2313757, ERS2317311, ERS2317312, ERS2317683, ERS2317684, ERS2318175 and ERS2318176.

**Dataset S1.** Measurements of dentitions

|  |  | Cusp number | | | | Top-cusp angle | | | |
| --- | --- | --- | --- | --- | --- | --- | --- | --- | --- |
| Species |  | P2 | P3 | P4 | P5 | P2 | P3 | P4 | P5 |
| *Halichoerus* X *Pusa* | A619266 | 3 | 4 | 4 | 4 | 69.4 | 71.8 | 93.2 | 109.5 |
| *Halichoerus* | 47754 | 1 | 1 | 3 | 3 | 40.5 | 47.7 | 71.7 | 87.3 |
|  | 47755 | 1 | 3 | 3 | 4 | - | - | - | 90.6 |
|  | 47757 | 2 | 2 | 3 | 3 | - | - | - | 92.0 |
|  | 47760 | 2 | 3 | 3 | 3 | - | 62.8 | - | 88.7 |
|  | 47761 | 1 | 2 | 2 | 4 | - | - | 69.4 | - |
|  | 47762 | 1 | 1 | 3 | 3 | - | 47.7 | - | - |
|  | 47763 | 1 | 2 | 3 | 3 | - | - | 76.1 | 96.9 |
|  | 47769 | 1 | 3 | 3 | 3 | 46.6 | 63.0 | 81.4 | - |
|  | 47771 | 2 | 3 | 3 | 3 | - | 65.1 | - | 96.7 |
|  | 47797 | 1 | 1 | 3 | 3 | - | - | - | - |
|  | 47798 | 1 | 1 | 3 | 2 | 43.4 | - | 77.6 | 84.2 |
|  | 47819 | 2 | 3 | 3 | 3 | - | 58.1 | 81.9 | 94.0 |
|  | 47820 | 2 | 2 | 3 | 2 | 58.7 | - | 78.3 | 89.3 |
|  | 47869 | 1 | 1 | 3 | 3 | - | 47.5 | 77.3 | 87.9 |
|  | 47886 | 1 | 3 | 3 | 3 | 40.9 | - | - | 96.0 |
|  | 47888 | 1 | 3 | 3 | 3 | 47.7 | 57.4 | - | - |
|  | 47889 | 1 | 3 | 3 | 3 | 48.2 | 60.3 | 82.5 | 99.7 |
|  | 47919 | 1 | 3 | 3 | 4 | 40.8 | 56.4 | - | - |
|  | 47931 | 2 | 2 | 3 | 3 | 57.5 | - | 77.8 | 87.6 |
|  | 47940 | 1 | 1 | 3 | 2 | 44.4 | 42.3 | 73.5 | 84.2 |
|  | 47942 | 2 | 3 | 3 | 4 | - | 63.2 | 73.4 | - |
|  | 47946 | 1 | 1 | 2 | 1 | 44.3 | 45.4 | - | 62.3 |
|  | 48119 | 1 | 1 | 3 | 3 | - | - | - | 93.5 |
|  | 48120 | 1 | 1 | 3 | 2 | 50.2 | 47.7 | 77.1 | 85.6 |
|  | 48128 | 1 | 1 | 3 | 3 | 38.6 | 45.7 | - | 94.9 |
|  | 48130 | 1 | 1 | 3 | 3 | - | 46.6 | - | 96.5 |
|  | 48132 | 2 | 3 | 3 | 3 | - | - | - | 103.9 |
|  | 48133 | 2 | 2 | 3 | 3 | - | - | - | 90.3 |
|  | 48145 | 1 | 2 | 2 | 3 | 42.1 | 63.4 | 72.5 | 90.0 |
|  | 48146 | 2 | 1 | 3 | 3 | 52.6 | 43.5 | 69.7 | 90.2 |
|  | 48147 | 1 | 1 | 3 | 3 | 41.4 | 46.7 | - | 91.7 |
|  | 48149 | 1 | 3 | 3 | 3 | 40.5 | 62.6 | 71.8 | 92.0 |
|  | 48159 | 1 | 1 | 3 | 3 | - | - | 84.1 | - |
|  | 48164 | 1 | 1 | 2 | 2 | 40.0 | - | 67.9 | 84.7 |
|  | 48188 | 1 | 1 | 3 | 3 | 40.6 | 43.0 | 73.3 | 95.5 |
|  | 48189 | 1 | 1 | 3 | 3 | 42.2 | 43.1 | 70.8 | 94.8 |
|  | 48193 | 1 | 3 | 3 | 3 | - | 63.1 | 73.5 | 101.7 |
|  | 48195 | 1 | 1 | 3 | 3 | 41.1 | - | 76.4 | 93.3 |
|  | 48250 | 1 | 1 | 3 | 3 | - | 46.8 | 80.6 | - |
|  | 48263 | 2 | 3 | 3 | 5 | 42.1 | 61.3 | 81.1 | 97.0 |
|  | 48299 | 1 | 1 | 3 | 3 | 47.3 | 47.4 | - | - |
|  | 48300 | 1 | 3 | 3 | 3 | - | - | - | 94.2 |
|  | 48310 | 2 | 3 | 3 | 3 | - | 66.8 | 82.0 | - |
|  | 48317 | 1 | 1 | 3 | 2 | 44.2 | 48.3 | 75.3 | 70.3 |
|  | 48319 | 1 | 2 | 2 | 3 | 46.5 | 62.0 | 68.5 | 91.5 |
|  | 48356 | 1 | 2 | 3 | 3 | 40.9 | - | 75.6 | 94.2 |
|  | 48358 | 1 | 2 | 3 | 2 | 40.3 | 59.6 | 68.6 | 80.0 |
|  | 48360 | 1 | 1 | 3 | 2 | 46.0 | 50.3 | 71.8 | 80.6 |
|  | 48361 | 1 | 3 | 3 | 3 | 41.7 | 62.6 | 81.5 | 100.3 |
|  | 48525 | 1 | 1 | 3 | 4 | 41.4 | - | 74.8 | 102.2 |
|  | 48670 | 1 | 2 | 3 | 3 | 42.1 | - | 76.9 | 89.8 |
|  | 48702 | 1 | 1 | 2 | 3 | 39.6 | 44.9 | 73.8 | 97.1 |
|  | 48706 | 1 | 2 | 3 | 2 | 38.8 | 56.9 | 73.4 | 78.9 |
|  | 48712 | 1 | 1 | 2 | 2 | 37.4 | - | - | 77.8 |
|  | 48720 | 1 | 2 | 2 | 2 | 47.8 | 62.0 | 75.5 | 93.3 |
|  | 48724 | 1 | 1 | 3 | 3 | 38.6 | 43.5 | - | 106.5 |
|  | 48726 | 1 | 2 | 3 | 3 | 42.0 | 60.4 | - | 95.8 |
|  | HG-01 | 1 | 1 | 3 | 3 | 46.5 | 49.6 | 78.1 | 96.9 |
|  | HG-03 | 2 | 2 | 2 | 2 | 58.6 | 62.3 | 68.2 | 86.8 |
|  | HG-04 | 1 | 2 | 3 | 2 | 45.6 | 63.4 | - | 68.6 |
|  | HG-11 | 1 | 3 | 3 | 3 | - | - | 80.0 | 107.0 |
|  | HG-13 | 3 | 2 | 3 | 3 | - | 59.6 | - | 88.8 |
|  | HG-15 | 1 | 1 | 3 | 2 | 40.7 | 44.7 | - | 83.7 |
|  | HG-16 | 1 | 1 | 3 | 3 | - | 45.0 | - | 91.1 |
|  | HG-17 | 1 | 1 | 2 | 3 | - | 48.2 | - | - |
| *Pusa* | 1149 | 4 | 4 | 4 | 3 | 97.8 | 106.1 | 113.5 | 120.6 |
|  | 1151 | 4 | 4 | 4 | 4 | 81.7 | 97.8 | - | 112.2 |
|  | 1157 | 4 | 4 | 4 | 3 | - | 103.8 | 105.3 | 122.6 |
|  | 1164 | 4 | 4 | 4 | 4 | - | 107.7 | 119.0 | - |
|  | 1167 | 4 | 4 | 4 | 3 | 89.9 | - | 118.0 | 121.1 |
|  | 1170 | 4 | 4 | 4 | 4 | 86.3 | 103.8 | 108.0 | 109.7 |
|  | 1178 | 4 | 4 | 4 | 4 | - | 89.5 | 108.7 | 112.8 |
|  | 1601 | 4 | 4 | 4 | 4 | 84.5 | 105.0 | 111.4 | 122.5 |
|  | 2264 | 4 | 4 | 3 | 3 | 111.1 | 110.5 | - | 137.8 |
|  | 2265 | 4 | 4 | 4 | 4 | 99.5 | 101.9 | 116.9 | 127.5 |
|  | 2273 | 4 | 4 | 4 | 4 | - | 110.7 | 115.7 | - |
|  | 2274 | 4 | 4 | 4 | 3 | 101.8 | 113.5 | 123.2 | 131.8 |
|  | 2275 | 4 | 4 | 4 | 4 | 101.6 | 112.0 | 116.1 | 122.3 |
|  | 2280 | 4 | 5 | 4 | 4 | - | 117.1 | 116.9 | 126.5 |
|  | 2281 | 4 | 4 | 4 | 4 | 100.6 | 105.3 | 104.5 | 125.8 |
|  | 2283 | 4 | 5 | 5 | 4 | 106.4 | 109.0 | - | 127.6 |
|  | 2287 | 4 | 4 | 5 | 5 | 92.1 | 96.2 | - | - |
|  | 2289 | 4 | 4 | 5 | 4 | - | - | 127.6 | 139.1 |
|  | 2306 | 4 | 4 | 4 | 4 | - | - | 113.1 | 116.6 |
|  | 2307 | 4 | 4 | 4 | 4 | - | - | - | 134.2 |
|  | 3660 | 4 | 5 | 5 | 5 | 98.3 | 107.0 | - | - |
|  | 3661 | 4 | 4 | 4 | 4 | 111.7 | 115.4 | - | - |
|  | 6739 | 4 | 4 | 4 | 4 | - | - | - | 121.0 |
|  | 39599 | 4 | 4 | 4 | 4 | - | 98.3 | 109.8 | 118.2 |
|  | 39782 | 4 | 4 | 4 | 3 | 104.0 | - | - | 124.3 |
|  | 47154 | 4 | 4 | 4 | 4 | 92.8 | 98.5 | 106.9 | 115.9 |
|  | 47157 | 4 | 4 | 4 | 3 | - | 103.9 | 106.3 | 123.4 |
|  | 47165 | 4 | 5 | 5 | 3 | - | 113.9 | 118.0 | 122.2 |
|  | 47166 | 4 | 4 | 4 | 4 | 103.0 | 107.6 | 131.9 | 121.3 |
|  | 47168 | 4 | 4 | 4 | 4 | 101.3 | - | 108.5 | 112.5 |
|  | 47170 | 4 | 4 | 4 | 5 | - | 95.2 | 102.8 | - |
|  | 47171 | 4 | 4 | 4 | 3 | 100.1 | 106.0 | 108.3 | 116.6 |
|  | 47173 | 4 | 4 | 4 | 3 | 98.0 | 102.9 | 111.0 | - |
|  | 47174 | 4 | 4 | 4 | 3 | - | - | 111.6 | 122.1 |
|  | 47178 | 4 | 4 | 4 | 3 | 106.1 | 112.0 | - | 122.6 |
|  | 47179 | 4 | 5 | 5 | 4 | 107.8 | 111.0 | 126.9 | 113.6 |
|  | 47182 | 4 | 4 | 4 | 4 | 104.0 | 113.8 | 116.2 | 130.8 |
|  | 47187 | 4 | 4 | 5 | 3 | 91.8 | 105.3 | 110.4 | 116.9 |
|  | 47188 | 4 | 4 | 4 | 3 | - | 95.0 | 107.3 | - |
|  | 47194 | 4 | 4 | 4 | 3 | 101.1 | 102.2 | 99.4 | 126.4 |
|  | 47195 | 4 | 4 | 4 | 4 | 94.3 | 95.6 | 106.3 | 117.0 |
|  | 47196 | 4 | 4 | 4 | 4 | 100.9 | 106.6 | - | 120.5 |
|  | 47198 | 4 | 4 | 5 | 5 | 94.0 | 91.2 | 105.6 | - |
|  | 47199 | 4 | 4 | 4 | 4 | 90.0 | 95.1 | 101.1 | 123.6 |
|  | 47200 | 4 | 5 | 5 | 5 | 109.6 | 110.0 | 121.6 | 134.4 |
|  | 47202 | 4 | 5 | 5 | 5 | 112.0 | 113.6 | 127.6 | 127.0 |
|  | 47203 | 4 | 4 | 5 | 5 | 95.1 | 109.4 | 117.3 | 133.6 |
|  | 47205 | 4 | 4 | 4 | 4 | - | - | - | - |
|  | 47240 | 4 | 4 | 4 | 3 | 95.2 | 95.9 | 105.0 | 111.3 |
|  | 47243 | 4 | 4 | 4 | 3 | - | 108.8 | - | - |
|  | 47246 | 4 | 4 | 4 | 3 | 103.8 | 115.3 | 111.7 | 116.3 |
|  | 47248 | 4 | 4 | 4 | 3 | 91.3 | 95.8 | 107.8 | 117.7 |
|  | 47250 | 4 | 4 | 5 | 5 | - | - | 109.7 | 130.3 |
|  | 47262 | 4 | 4 | 4 | 4 | - | 109.7 | 110.8 | 120.7 |
|  | 47263 | 4 | 4 | 4 | 4 | - | - | 105.7 | - |
|  | 47267 | 4 | 4 | 4 | 3 | 96.4 | 108.1 | 111.9 | - |
|  | 47273 | 4 | 4 | 4 | 3 | 99.2 | 105.8 | - | - |
|  | 47277 | 4 | 4 | 4 | 4 | 90.2 | - | 110.3 | 124.2 |
|  | 47281 | 4 | 4 | 4 | 3 | 107.7 | - | 118.3 | 113.8 |
|  | 47283 | 4 | 4 | 4 | 3 | 91.2 | - | 98.4 | - |
|  | 47286 | 4 | 4 | 4 | 3 | - | - | - | 110.2 |
|  | 47304 | 5 | 5 | 5 | 5 | 103.3 | 112.3 | 111.4 | 128.9 |
|  | 47309 | 4 | 4 | 4 | 3 | 103.7 | 111.7 | 114.4 | 131.8 |
|  | 47325 | 4 | 4 | 4 | 4 | 97.7 | 111.4 | 116.4 | - |
|  | 47342 | 4 | 4 | 4 | 4 | - | 113.5 | - | 127.5 |

Specimen numbers denote to Swedish museum of Natural history (the hybrid), Finnish Museum of Natural History and Saimaa Ringed Seal Genome Project (numbers beginning with a letter). Data are from the right side. Cusp have been tabulated regardless how large they are. Missing angle measurements are for teeth with cracked or worn cusp tips. P2 to P5 refers to second to fifth postcanine teeth.

**Dataset S2.** Skull PCA data

| Species | Specimen | Age | Sex | PC1 | PC2 | PC3 |
| --- | --- | --- | --- | --- | --- | --- |
| *Halichoerus* X *Pusa* | A619266 | j | f | 0.03291 | 0.04504 | -0.03272 |
|  | Juvenile mean | j | - | 0.02776 | 0.03654 | -0.00270 |
| *Halichoerus* | 2240 | j | f | -0.01119 | 0.04626 | -0.01334 |
|  | 47755 | j | f | 0.00773 | 0.0532 | 0.00693 |
|  | 48159 | j | f | 0.00257 | 0.04493 | 0.00823 |
|  | 48261 | j | f | 0.0098 | 0.01988 | 0.02379 |
|  | 48300 | j | f | -0.00146 | 0.05509 | 0.01465 |
|  | 47757 | j | m | -0.00776 | 0.05966 | 0.00849 |
|  | 47770 | j | m | -0.01154 | 0.05394 | 0.00011 |
|  | 47771 | j | m | -0.00706 | 0.07205 | -0.01213 |
|  | 47773 | j | m | -0.0039 | 0.02958 | 0.04761 |
|  | 47890 | j | m | 0.0164 | 0.04344 | 0.00865 |
|  | 48263 | j | m | -0.01262 | 0.03304 | -0.00652 |
|  | 2311 | a | f | -0.07431 | 0.00786 | -0.00373 |
|  | 2315 | a | f | -0.0799 | 0.00835 | -0.01556 |
|  | 23104 | a | f | -0.07192 | -0.01784 | -0.00887 |
|  | 47763 | a | f | -0.0079 | 0.01142 | 0.01808 |
|  | 47798 | a | f | -0.01049 | 0.01799 | 0.02437 |
|  | 47800 | a | f | -0.0015 | -0.00011 | 0.04086 |
|  | 47888 | a | f | -0.01019 | 0.00703 | 0.00726 |
|  | 47919 | a | f | -0.0169 | -0.01165 | 0.01935 |
|  | 47924 | a | f | -0.03681 | -0.02106 | 0.01764 |
|  | 47926 | a | f | -0.05077 | 0.01289 | -0.01612 |
|  | 47931 | a | f | -0.00205 | 0.01124 | 0.0249 |
|  | 47942 | a | f | 0.00274 | 0.01279 | 0.0398 |
|  | 47946 | a | f | -0.02446 | 0.01096 | 0.01908 |
|  | 48120 | a | f | -0.00605 | 0.0342 | 0.01013 |
|  | 48128 | a | f | -0.0302 | -0.01761 | 0.02587 |
|  | 48146 | a | f | -0.04521 | -0.00865 | 0.01092 |
|  | 48147 | a | f | -0.0471 | -0.00973 | 0.01581 |
|  | 48149 | a | f | -0.04038 | -0.01465 | 0.022 |
|  | 48153 | a | f | -0.08782 | -0.00552 | -0.01381 |
|  | 48154 | a | f | -0.03078 | -0.00569 | 0.02002 |
|  | 48164 | a | f | -0.0414 | -0.00036 | 0.01494 |
|  | 48193 | a | f | -0.04351 | -0.01103 | 0.0068 |
|  | 48278 | a | f | -0.05115 | -0.01236 | -0.00545 |
|  | 48296 | a | f | -0.07364 | -0.00765 | -0.0159 |
|  | 48356 | a | f | -0.00961 | 0.00621 | 0.02772 |
|  | 48361 | a | f | -0.02895 | -0.01211 | 0.0161 |
|  | 48525 | a | f | -0.02609 | 0.00949 | 0.02762 |
|  | 48670 | a | f | -0.01186 | -0.00183 | 0.03451 |
|  | 48712 | a | f | -0.06168 | -0.01071 | 0.00966 |
|  | 4450 | a | m | -0.12015 | -0.00049 | -0.05881 |
|  | 6509 | a | m | -0.11419 | 0.00709 | -0.06157 |
|  | 6563 | a | m | -0.08649 | -0.01963 | 0.00757 |
|  | 47761 | a | m | -0.01319 | 0.00706 | 0.01561 |
|  | 47769 | a | m | -0.02354 | 0.00646 | 0.01828 |
|  | 47788 | a | m | -0.01362 | 0.00505 | 0.0331 |
|  | 47794 | a | m | 0.0018 | 0.013 | 0.02232 |
|  | 47797 | a | m | -0.03687 | -0.01109 | 0.01096 |
|  | 47802 | a | m | 0.00194 | 0.02804 | 0.00864 |
|  | 47883 | a | m | -0.08613 | -0.00462 | -0.00597 |
|  | 47936 | a | m | -0.01782 | 0.00197 | 0.01411 |
|  | 47940 | a | m | -0.00235 | 0.01173 | 0.00381 |
|  | 48119 | a | m | 0.00655 | 0.02698 | 0.01109 |
|  | 48133 | a | m | -0.02139 | 0.01194 | 0.00993 |
|  | 48158 | a | m | -0.10277 | -0.00469 | -0.03253 |
|  | 48172 | a | m | -0.11243 | -0.00743 | -0.01291 |
|  | 48250 | a | m | -0.05845 | -0.01197 | 0.00865 |
|  | 48286 | a | m | -0.06053 | -0.00603 | -0.01796 |
|  | 48299 | a | m | -0.0755 | -0.03945 | 0.0058 |
|  | 48319 | a | m | -0.07837 | 0.01459 | -0.01928 |
|  | 48343 | a | m | -0.09879 | -0.01288 | -0.01964 |
|  | 48360 | a | m | -0.01943 | -0.00834 | 0.04435 |
|  | 48720 | a | m | -0.05392 | -0.0003 | -0.00064 |
|  | 48724 | a | m | -0.10003 | 0.00745 | -0.03367 |
|  | 48725 | a | m | -0.01254 | -0.01267 | 0.02334 |
|  | 48726 | a | m | -0.04834 | -0.01382 | -0.00663 |
| *Pusa* | 2286 | j | f | 0.07207 | 0.04822 | -0.01444 |
|  | 2288 | j | f | 0.05305 | 0.03041 | -0.03387 |
|  | 47162 | j | f | 0.06969 | 0.02396 | -0.0099 |
|  | 47245 | j | f | 0.07939 | 0.01989 | -0.01366 |
|  | 47252 | j | f | 0.0459 | 0.00991 | -0.0018 |
|  | 47259 | j | f | 0.0584 | 0.00534 | -0.00555 |
|  | 47309 | j | f | 0.05725 | 0.01129 | -0.02217 |
|  | 558 | j | m | 0.06096 | 0.0411 | -0.02339 |
|  | 47246 | j | m | 0.07404 | 0.01492 | -0.01719 |
|  | 47253 | j | m | 0.0586 | 0.01999 | -0.02223 |
|  | 47306 | j | m | 0.055 | 0.02205 | 0.0089 |
|  | 47155 | a | f | 0.02495 | -0.04085 | -0.01556 |
|  | 47180 | a | f | 0.04956 | -0.03522 | 0.0071 |
|  | 47187 | a | f | 0.06311 | -0.00956 | -0.03385 |
|  | 47194 | a | f | 0.03941 | -0.01517 | -0.01241 |
|  | 47196 | a | f | 0.06828 | -0.01174 | 0.00368 |
|  | 47197 | a | f | 0.06782 | 0.00033 | -0.01818 |
|  | 47200 | a | f | 0.04252 | -0.03906 | -0.01426 |
|  | 47204 | a | f | 0.04564 | -0.03086 | 0.00363 |
|  | 47243 | a | f | 0.02921 | -0.02759 | -0.0068 |
|  | 47247 | a | f | 0.03985 | -0.00751 | -0.02507 |
|  | 47248 | a | f | 0.05506 | -0.00177 | -0.00438 |
|  | 47267 | a | f | 0.04236 | -0.02441 | -0.00356 |
|  | 47268 | a | f | 0.02585 | -0.03514 | -0.00169 |
|  | 47285 | a | f | 0.03276 | -0.02714 | -0.02334 |
|  | 47295 | a | f | 0.06451 | -0.02254 | -0.00309 |
|  | 47305 | a | f | 0.04785 | -0.02944 | -0.00588 |
|  | 47324 | a | f | 0.03012 | -0.03514 | -0.02014 |
|  | 47438 | a | f | 0.05727 | -0.03239 | 0.03426 |
|  | 47159 | a | m | 0.0518 | -0.00262 | -0.00724 |
|  | 47160 | a | m | 0.00871 | -0.0452 | 0.01395 |
|  | 47166 | a | m | 0.03948 | -0.03881 | 0.00627 |
|  | 47201 | a | m | 0.04251 | -0.01836 | -0.01052 |
|  | 47205 | a | m | 0.0589 | 0.00654 | -0.01146 |
|  | 47240 | a | m | 0.06382 | 0.0212 | -0.01818 |
|  | 47250 | a | m | 0.0618 | -0.011 | 0.00245 |
|  | 47274 | a | m | 0.05916 | 0.01116 | -0.00256 |
|  | 47276 | a | m | 0.05375 | -0.0356 | 0.00599 |
|  | 47277 | a | m | 0.05709 | -0.01639 | 0.00048 |
|  | 47291 | a | m | 0.04618 | -0.0167 | -0.00676 |
|  | 47300 | a | m | 0.04033 | -0.02545 | -0.00194 |
|  | 47304 | a | m | 0.04776 | -0.02687 | 0.00686 |
|  | 47308 | a | m | 0.0083 | -0.0349 | -0.02271 |
|  | 47314 | a | m | 0.03556 | -0.03172 | 0.01405 |
|  | 47325 | a | m | 0.01167 | -0.0423 | -0.00333 |
|  | 47342 | a | m | 0.04154 | -0.01315 | -0.02846 |
|  | 47343 | a | m | 0.02212 | -0.03183 | -0.02299 |
|  | 47363 | a | m | 0.01584 | -0.02266 | -0.00693 |
|  | 47365 | a | m | 0.04794 | -0.00969 | -0.00514 |

Specimen numbers denote to Swedish museum of Natural history (the hybrid) and Finnish Museum of Natural History. Categories listed are juvenile (j) adult (a), female (f), and male (m).

Movie S1. Main patterns of allometric growth for ringed and grey seals represented as a series of predicted shapes from newborns to adults computed from quadratic multivariate regressions of shape onto centroid size. Visualisations obtained via morphing of 3D surface scans of newborns using Thin Plate Spline (TPS). Same as in figure 3.
